# A brain-first framework for perimenopause management: the case for non-invasive neuromodulation

**DOI:** 10.3389/fgwh.2026.1868506

**Published:** 2026-07-06

**Authors:** Emilė Radytė, Ieva Karvelyte

**Affiliations:** Samphire Neuroscience, London, United Kingdom

**Keywords:** cognition, menopause, mood disorders, perimenopause, transcranial direct current stimulation, women’s health

## Abstract

The menopause transition affects approximately half the global population yet remains substantially under-researched and under-treated. Standard care is dominated by menopausal hormone therapy (MHT), with adjunctive use of selective serotonin and noradrenaline reuptake inhibitors and, more recently, neurokinin-3 receptor antagonists. While these approaches have transformed care for vasomotor symptoms, they leave significant unmet needs across cognitive, affective, and sleep-related domains, and remain unsuitable or undesirable for many women. In this Perspective, we argue for a complementary brain-first framework for perimenopause management. Four converging lines of evidence support this framing: perimenopause is fundamentally a loss of cyclical predictability rather than a gradual hormone deficiency, with progesterone-driven loss of inhibitory neurosteroidal tone preceding estradiol variability; vasomotor symptoms originate centrally in hypothalamic kisspeptin/neurokinin B/dynorphin circuits; the perimenopausal brain undergoes measurable structural, metabolic, and network-level reorganization; and these brain-mediated changes can precede and outlast peripheral hormonal markers, with marked inter-individual variability that systemic hormone levels alone do not capture. Within this framework, we examine non-invasive brain stimulation—specifically transcranial direct current stimulation (tDCS)—as a candidate brain-first intervention. We synthesize existing tDCS evidence relevant to perimenopausal symptom clusters, identify gaps in dedicated trials, and outline future research priorities, including outcome measures, study populations, and integration with digital health platforms. The brain-first framing is offered as complementary to, not in place of, established hormonal and pharmacological approaches.

## 1 Introduction

The menopause transition is among the most universal experiences in women's health, yet remains one of the most under-served. By 2030, the global population of menopausal and post-menopausal women is projected to exceed 1.2 billion (1). Symptom burden during the perimenopause and early post-menopause is substantial: vasomotor symptoms (VMS) affect up to 80% of women, with frequent VMS persisting a median of 7.4 years and over 4 years post-final menstrual period in the Study of Women's Health Across the Nation (SWAN) cohort (2). Cognitive complaints, mood disturbance, and sleep fragmentation affect a similar proportion and contribute substantially to reduced quality of life (3). Despite this prevalence, perimenopause-specific research and clinical training have lagged comparable areas of medicine for decades, and recent commentary has highlighted both the unmet needs of women across the transition and the limitations of framing menopause solely as a hormone-deficiency disorder (1).

Current management centers on menopausal hormone therapy (MHT). MHT is effective for vasomotor and urogenital symptoms and has favorable long-term effects on bone density (1). Despite this, MHT use has declined substantially over two decades, from 26.9% of US postmenopausal women in 1999 to 4.7% in 2017–2020 (4). However, it is contraindicated in subsets of women—for example, those with hormone-sensitive malignancies, certain thrombotic risks, or unmanaged cardiovascular disease—and is declined by many others on grounds of preference, perceived risk, or access; perceptions were shaped in part by FDA boxed warnings revised in 2025 to reflect contemporary evidence (5). Non-hormonal alternatives, such as selective serotonin and noradrenaline reuptake inhibitors, gabapentinoids, clonidine, and most recently the neurokinin-3 receptor antagonist fezolinetant, have expanded the toolkit but address only a subset of symptoms and carry their own tolerability constraints (6).

Among non-invasive brain stimulation modalities, transcranial direct current stimulation (tDCS) is the most tractable candidate for perimenopausal care, owing to its portability, established safety record, and feasibility of self-administered home use. tDCS delivers low-amplitude direct current (typically 1–2 mA) via scalp electrodes, shifting the resting membrane potential of cortical neurons and modulating spontaneous firing rates without triggering discrete action potentials (7). Anodal stimulation over the dorsolateral prefrontal cortex (DLPFC)—its most extensively studied target—increases cortical excitability and engages prefrontal–limbic circuits that govern mood regulation, executive function, sleep, and pain modulation (8, 9). As we develop in Section 2, perimenopausal cognitive, affective, and sleep symptoms are correlated with neuroimaging-detected changes in these same prefrontal–limbic circuits that co-occur with the loss of estrogenic and progesterone-derived neurosteroidal modulation; the evidence to date is associative rather than causal. The mechanistic hypothesis underlying tDCS as a brain-first intervention is that direct modulation of cortical excitability can partially compensate for this regulatory loss, offering a non-hormonal route to engaging the same neural substrate.

Compared with transcranial magnetic stimulation, tDCS uses lower-cost hardware and does not require specialist operators. Compared with transcranial alternating current stimulation, its evidence base in mood, pain, and sleep is more developed. A favourable safety profile across more than 33,200 sessions (7) and fully remote delivery (10) make tDCS well placed to address the access gap in perimenopause care (1). Throughout, “brain-first” denotes an approach that targets the central nervous system as a primary site for understanding and intervening in perimenopausal symptoms, complementing rather than replacing peripheral-hormonal frameworks.

This Perspective is narrative rather than systematic; we identified evidence through targeted PubMed and Cochrane searches in each domain, prioritising meta-analyses, evidence-based guidelines, and large cohort or imaging studies, and cite work that is illustrative rather than exhaustive.

## 2 The brain at the center of the menopause transition

The menopause transition is, fundamentally, a neuroendocrine event. Four converging strands of evidence support a brain-first framing.

### 2.1 Perimenopause is a loss of cyclical predictability, not a gradual hormone deficiency

A persistent misconception in clinical practice is that perimenopause is characterized by a smooth, unidirectional decline in estrogen. Longitudinal studies of daily and serial hormonal sampling demonstrate the opposite: perimenopause is defined by *erratic variability* rather than steady decline, with estradiol swinging between supraphysiological peaks and deep troughs across and within cycles, and average levels often remaining within reproductive-age reference ranges well into the early transition (11). One immediate clinical consequence is that single-timepoint serum hormone measurements are unreliable for diagnosing perimenopause (11). A more fundamental consequence is that the brain—which evolved to operate against a backdrop of predictable cyclical signaling—must adapt to an increasingly noisy hormonal environment.

Within this disrupted predictability, the earliest changes often involve progesterone. Luteal-phase progesterone production declines as ovulatory cycles become less consistent (11). Because progesterone metabolizes to allopregnanolone, a GABA-A positive allosteric modulator, its loss reduces inhibitory neurosteroidal tone and contributes to anxiety, sleep fragmentation, and emotional reactivity in early perimenopause (3). Estradiol variability follows, with rising FSH and LH reflecting reduced ovarian responsiveness (11).

### 2.2 Vasomotor symptoms originate in central thermoregulatory circuits

The most prevalent menopausal symptom—the hot flush—originates in hypothalamic kisspeptin/neurokinin B/dynorphin (KNDy) neurons. In the absence of estrogenic restraint, these neurons hypertrophy and release neurokinin B onto thermoregulatory targets in the median preoptic nucleus, triggering the cutaneous vasodilation and sweating that characterize the hot flush (12, 13). The clinical effectiveness of fezolinetant, a selective neurokinin-3 receptor antagonist that acts directly on this central circuit, provides translational validation that VMS are a centrally-generated phenomenon rather than a peripheral consequence of hormone withdrawal: in the SKYLIGHT 1 phase III trial, fezolinetant produced significant reductions in moderate-to-severe VMS frequency and severity by week 4 and sustained through week 12 (6). Direct tDCS evidence is limited to one small sham-controlled trial in postmenopausal women that did not show efficacy on VMS [(14); see Section 4]; no tDCS study has targeted the hypothalamic VMS circuit specifically.

### 2.3 The brain undergoes structural, metabolic, and network reorganization

Beyond VMS, the perimenopause-to-menopause transition is associated with measurable changes in brain structure, energy metabolism, and large-scale network organization that map onto the cognitive and affective symptom profile.

#### 2.3.1 Prefrontal-limbic regulation becomes less efficient

Estrogen receptors are densely distributed across cortical, hippocampal, and limbic regions, where estradiol modulates synaptic plasticity and prefrontal-limbic connectivity (3); with the loss of GABAergic restraint from declining progesterone metabolites (Section 2.1), this contributes to the emotional reactivity and impaired executive function reported across longitudinal studies. Direct evidence comes from multimodal neuroimaging documenting transition-stage changes in prefrontal-limbic structure and connectivity (15); the “less efficient” characterisation reflects this together with the behavioural findings below, since task-based fMRI in perimenopause remains limited. SWAN data show verbal episodic memory and processing speed fail to gain the expected practice-related improvement during perimenopause, with partial rebound in postmenopause (16); STRAW+10-staged work shows decrements in working memory and verbal learning most evident in the late transition (17). The International Menopause Society concludes these cognitive changes are real and not explained by age or mood alone (3).

#### 2.3.2 Cerebral energy metabolism shifts

Estradiol regulates cerebral glucose metabolism, mitochondrial function, and ATP generation (18), and its decline produces a measurable bioenergetic transition: 18F-fluorodeoxyglucose positron emission tomography across STRAW+10-staged endocrine groups shows progressive declines in cerebral glucose metabolic rate in Alzheimer's-vulnerable regions, correlated with reduced mitochondrial cytochrome oxidase activity and emerging at perimenopause rather than postmenopause (19). This bioenergetic transition provides a mechanistic substrate for commonly reported cognitive symptoms—brain fog, word-finding difficulty, reduced processing speed—and for the coexisting fatigue and autonomic hyperarousal characteristic of perimenopause.

#### 2.3.3 Brain networks reorganize

Multimodal neuroimaging across the menopause transition documents gray matter loss, white matter alterations, reduced cerebral glucose metabolism, and emerging amyloid-beta deposition in APOE-ε4 carriers—effects independent of chronological age, distinct from those in age-matched men, and partially reversing in late post-menopause alongside cognitive performance (15). Estrogen-sensitive hub networks, especially the default mode network, show reduced connectivity stability across the transition (15), a candidate correlate for the disrupted mental continuity women commonly describe.

### 2.4 Brain changes can precede and outlast peripheral hormonal markers, and vary substantially between women

Subjective cognitive and affective symptoms often emerge early in the menopause transition, when serum hormonal markers may still appear within reproductive-age reference ranges, and can persist into postmenopause despite hormonal stabilization (3, 11, 16). Neuroimaging biomarkers similarly track menopausal endocrine stage rather than chronological age, and only partially reverse after peripheral stabilization (15). This temporal mismatch argues against a model in which the brain is a passive downstream target of ovarian decline, and in favor of one in which the brain is an active, partially independent locus of the menopause transition.

Equally striking is the inter-individual variability of symptom severity. While estradiol and progesterone decline along broadly comparable trajectories across women, neurosteroidal compensation does not: adrenal-derived dehydroepiandrosterone (DHEA) and its sulphate (DHEAS) are brain-synthesized neurosteroids that modulate GABA-A and NMDA receptor function and serve as local precursors for estradiol and androgen production (20). DHEAS levels vary with age, chronic stress, steroidogenic-enzyme polymorphisms, and reproductive history (20), providing a plausible substrate for variability in cognitive and affective outcomes. Responses to systemic hormone replacement vary similarly between women, reinforcing the argument for therapeutic strategies that act directly on the brain.

Taken together, these strands suggest that effective perimenopause care will benefit from interventions that act directly on the relevant neural circuits, complementing systemic hormonal approaches rather than depending exclusively on them.

## 3 Gaps in current management

Despite expansion of the perimenopause toolkit — menopausal hormone therapy (MHT), SSRIs/SNRIs, gabapentinoids, clonidine, and the neurokinin-3 receptor antagonist fezolinetant (Table 1) — three gaps persist within current care models.

**Table 1:** Comparison of current and investigational approaches to perimenopausal symptom management.

Modality	Main target symptoms	Proposed mechanism	Evidence in target indication	Status in perimenopause
Menopausal hormone therapy (MHT)	VMS, urogenital, bone health	Restoration of estrogenic tone	Established RCT and cohort evidence (1)	First-line where indicated; contraindicated in subsets
SSRIs/SNRIs	VMS, mood	Serotonergic and noradrenergic modulation	Moderate; off-label for VMS	Established adjunct; tolerability constraints (3)
Gabapentinoids, clonidine	VMS, sleep	Mixed central actions	Modest; heterogeneous evidence	Adjunct options; sedation and dependence concerns
Fezolinetant (NK3R antagonist)	VMS	Selective blockade of hypothalamic KNDy circuit (12, 13)	Phase 3 RCT (SKYLIGHT 1) (6)	Approved 2023 for moderate-severe VMS
Transcranial direct current stimulation (tDCS)	Investigational across mood, cognition, sleep, pain, VMS	Modulation of cortical excitability in prefrontal-limbic (DLPFC) and motor (M1) circuits (7–9, 21)	Indirect from adjacent indications (8, 9, 21–24); one small sham-controlled VMS trial (14)	Investigational only; no menopause approval

First, cognitive and affective symptoms are inadequately addressed. Evidence on MHT effects on cognition in perimenopause remains mixed, with no consistent benefit to support routine prescription for cognitive complaints (3). SSRIs and SNRIs reduce VMS and treat overt mood disorders, but have limited benefit on subclinical mood symptoms or executive function, and their tolerability profile—sleep disruption, sexual side effects, weight change—overlaps with the symptoms women seek to relieve.

Second, current options offer little for women with overlapping symptom clusters—concurrent cognitive, mood, and sleep symptoms in the absence of severe VMS. Pharmacological strategies typically involve sequential trials with cumulative side-effect burden and limited evidence to guide sequencing.

Third, access remains uneven. Specialist menopause services are concentrated in high-income urban centers, and even within well-resourced systems, primary care provision of perimenopause care is variable in both quality and reach (1). Scalable, decentralized, and self-administered interventions would substantially extend access, aligning with recent calls for a broader range of non-pharmacological options in menopause care (1). tDCS, examined below, must itself be evaluated against these access criteria. CE-marked home-use devices retail at several hundred to a few thousand pounds or euros, are not routinely reimbursed by health systems or insurers for any indication, and impose out-of-pocket costs that risk limiting reach. Adherence in home-based depression trials has been high [over 85% of sessions completed under remote supervision; (10)], but data on continued use after trials end are scarce; long-term adherence in perimenopause is an open empirical question.

These gaps motivate consideration of complementary approaches that act centrally, address multiple symptom domains simultaneously, and are deliverable at scale.

## 4 Non-invasive brain stimulation as a brain-first intervention

We now turn to the evidence base for tDCS in indications relevant to perimenopausal symptom domains. Stimulation targets vary by domain: the dorsolateral prefrontal cortex (DLPFC) is the predominant target for mood and cognition and is also associated with modulation of the affective dimensions of pain (8, 9); for chronic pain itself, the primary motor cortex (M1) is the most-studied target, with the somatosensory cortex and other cortical sites studied less extensively (21). Typical protocols deliver 1–2 mA for 20–30 min per session, 5 days/week over 2–6 weeks (total contact time ∼10–15 h per course). The evidence across these domains is at an early but informative stage:

- **Mood.** A meta-analysis of 23 trials (1,092 participants) reported small but significant active-vs.-sham effects on response and remission (9); a more recent individual-patient-data meta-analysis (18 trials, 1,246 participants) reported a Hedges’ g of 0.24 favouring active tDCS, with moderate certainty of evidence (8). This is conventionally small (Cohen's d 0.2 = small, 0.5 = medium); SSRIs in adult depression sit around g = 0.30 and supervised exercise around g = 0.4–0.7.
- **Pain.** Chronic pain has been the most extensively studied indication for tDCS, with the primary motor cortex (M1) as the predominant target. European evidence-based guidelines assign anodal M1 tDCS a Level B recommendation (probable efficacy) in fibromyalgia and Level C (possible efficacy) in chronic neuropathic pain (21). A tDCS meta-analysis of fibromyalgia—a condition that overlaps demographically and symptomatically with perimenopause—found tentative evidence of analgesic effect with substantial study heterogeneity (22). DLPFC stimulation, less extensively studied in pain, appears more consistent in modulating affective rather than sensory pain dimensions (21).
- **Sleep.** A systematic review of brain stimulation for insomnia found modest but consistent effects of multi-session tDCS on subjective (Pittsburgh Sleep Quality Index, Insomnia Severity Index) and objective (polysomnographic sleep efficiency and total sleep time) sleep parameters, with the strongest effects reported in patients with comorbid depression and insomnia, where 20 sessions of bilateral DLPFC stimulation (2 mA, 20 min per session) improved both polysomnographic and self-reported measures (23, 24).
- **Home-based delivery.** A fully remote, sham-controlled phase 2 trial demonstrated feasibility, safety, and antidepressant efficacy of home-based tDCS for major depressive disorder outside specialist clinics (10).

As noted in Section 2.2, the only direct tDCS evidence in postmenopausal women is a single sham-controlled trial in 30 women that did not show efficacy on VMS frequency or severity (14); the strongest current case for brain-first stimulation in perimenopause therefore rests on the broader cognitive, affective, sleep-related, and pain-related symptom complex rather than on hot flushes alone. Across these adjacent indications, study heterogeneity in stimulation protocol, electrode montage, dose, session number, sham design, and adherence is substantial, and inter-individual variability in response is high; direct extrapolation to perimenopausal women is therefore not warranted in the absence of dedicated trials.

Formal health-economic evaluations of tDCS are limited. A French hospital costing study estimated the per-patient cost of a 15-session tDCS depression programme at approximately €1,556 (25), and the DISCO trial is the first prospective cost-utility evaluation in non-treatment-resistant depression, with results pending (26). No cost-effectiveness analysis has been published for any women's health or menopausal indication.

As noted in Section 3, the structural cost characteristics of tDCS—lower hardware costs, no specialist operator requirement, and feasibility of fully remote delivery (10)—support a plausible case for favourable cost-effectiveness at scale. Future perimenopause trials should test this through integrated health-economic substudies.

## 5 Discussion: research priorities

Translating the brain-first framework into evidence-based care requires investment across several fronts.

### 5.1 Dedicated perimenopause trials

Most existing tDCS evidence comes from non-perimenopausal samples. Trials specifically designed for perimenopausal populations where the menopausal stage is classified using the STRAW+10 system (27), inclusion of women across the transition, and stratification by hormonal status are needed. These trials should be adequately powered for the multi-symptom presentation typical of perimenopause rather than focused on single endpoints in isolation. Where feasible, trials should include active-comparator arms (e.g., MHT or fezolinetant for VMS; SSRIs for mood) alongside sham, to support direct effect-size comparison rather than indirect cross-trial benchmarking.

### 5.2 Outcome measures

The brain-first framing implies that outcome measures should capture the full range of brain-mediated symptoms—cognition, mood, sleep, and pain—alongside vasomotor outcomes. Patient-reported outcome measures validated in perimenopausal populations (such as the Menopause-Specific Quality of Life Questionnaire and the Greene Climacteric Scale), depression and anxiety scales (e.g., PHQ-9, HAM-D, GAD-7), neurocognitive batteries sensitive to executive function and verbal memory (e.g., Stroop, Trail Making Test, CANTAB) (17), validated sleep instruments (Pittsburgh Sleep Quality Index, Insomnia Severity Index) with actigraphy or polysomnography where feasible, and pain measures (Brief Pain Inventory, visual analogue scales) should be considered as a coherent package. Composite endpoints reflecting multi-domain improvement may be more clinically meaningful than single-symptom reductions, particularly given that many women experience overlapping symptom clusters that no single pharmacological agent fully addresses.

### 5.3 Mechanistic biomarkers

Where feasible, trials should incorporate mechanistic biomarkers, such as EEG measures of cortical excitability or network dynamics, neuroimaging where appropriate (15), and serial hormonal sampling to test whether tDCS effects relate to circuit-level changes hypothesized by the brain-first framework. Such measures also support stratification of likely responders, an unresolved problem in the broader tDCS literature where individual-patient-data analyses have so far identified few reliable moderators of response (8).

### 5.4 Equity, access, and patient-centered design

Brain-first interventions at home can extend access only if device design and digital interfaces are inclusive. Given the historical privilege of cisgender women in high-income countries in menopause research (1), co-design and equitable trial inclusion are essential to avoid replicating MHT access inequities.

### 5.5 Regulatory and data considerations

At-home neuromodulation devices for women's health applications fall within evolving regulatory frameworks. Sponsors should engage early with regulators on endpoint design, sham-controlled trial methodology, and human factors validation appropriate to perimenopausal users. Data privacy considerations particular to femtech, such as sensitive symptom and reproductive data, warrant explicit attention in study and platform design.

Safety in at-home delivery merits dedicated attention. Although tDCS has an established safety profile in supervised settings (7), self-administered use requires screening for contraindications (seizure history, intracranial metal, scalp pathology), monitoring of common minor events (skin irritation, tingling, headache, dizziness), screening for neuropsychiatric comorbidity (including bipolar mania risk), and device and interface design that supports correct electrode placement and adherence reporting.

### 5.6 Complementarity, not substitution

A brain-first framing does not displace MHT or other established treatments. Rather, it expands the conceptual and therapeutic space, offering tools for women who cannot or do not wish to use hormonal options, for symptom domains that hormones address incompletely (3), and for combination strategies whose effects may be additive. The clinically useful question is not “tDCS or MHT?” but “for whom, when, and in what combination?”.

## 6 Conclusion

The perimenopause transition is fundamentally a brain phenomenon, expressed across cognitive, affective, autonomic, and sleep-related domains (3, 15). A brain-first framing reorients management toward the relevant neural systems and creates space for non-pharmacological, non-hormonal, and patient-administered interventions. tDCS is a promising candidate, with indirect evidence from adjacent indications—depression (8, 9), fibromyalgia pain (22), insomnia (23, 24)—and an established safety profile (7), but only one small sham-controlled trial in postmenopausal women to date (14). Clinical utility, safety, cost-effectiveness, and acceptability in perimenopause require confirmation in adequately powered, sham-controlled, perimenopause-specific trials. The brain-first framing is offered as a research hypothesis rather than a near-term clinical alternative.

## Data Availability

This Perspective synthesises previously published, publicly available peer-reviewed literature; no datasets were generated or analysed. All cited sources are accessible through the references listed in the manuscript, predominantly via PubMed and the publishers' websites.
